# Current status of a helicopter transportation system on remote islands for patients undergoing mechanical thrombectomy

**DOI:** 10.1371/journal.pone.0245082

**Published:** 2021-01-19

**Authors:** Takeshi Hiu, Shimpei Morimoto, Ayaka Matsuo, Kei Satoh, Hiroaki Otsuka, Fumiya Kutsuna, Keisuke Ozono, Kosuke Hirayama, Chikaaki Nakamichi, Kazumi Yamasaki, Yuka Ogawa, Eri Shiozaki, Yoichi Morofuji, Ichiro Kawahara, Nobutaka Horie, Yohei Tateishi, Tomonori Ono, Wataru Haraguchi, Tsuyoshi Izumo, Akira Tsujino, Takayuki Matsuo, Keisuke Tsutsumi

**Affiliations:** 1 Department of Neurosurgery, National Hospital Organization Nagasaki Medical Center, Nagasaki, Japan; 2 Department of Neurosurgery, Nagasaki University Graduate School of Biomedical Sciences, Nagasaki, Japan; 3 Innovation Platform & Office for Precision Medicine, Nagasaki University Graduate School of Biomedical Sciences, Nagasaki University, Nagasaki, Japan; 4 Department of Immunology and Rheumatology, Nagasaki University Graduate School of Biomedical Sciences, Nagasaki, Japan; 5 Clinical Research Center, Nagasaki University Hospital, Nagasaki, Japan; 6 Department of Neurology, National Hospital Organization Nagasaki Medical Center, Nagasaki, Japan; 7 Department of Emergency, National Hospital Organization Nagasaki Medical Center, Nagasaki, Japan; 8 Clinical Research Center, National Hospital Organization Nagasaki Medical Center, Nagasaki, Japan; 9 Department of Neurology, Nagasaki University Graduate School of Biomedical Sciences, Nagasaki, Japan; Hungarian Academy of Sciences, HUNGARY

## Abstract

**Background:**

Mechanical thrombectomy (MT) is standard treatment for acute ischemic stroke (AIS) with large-vessel occlusion within 6 h of symptom onset to treatment initiation (OTP). Recent trials have extended the therapeutic time window for MT to within 24 h. However, MT treatment remains low in remote areas. Nagasaki Prefecture, Japan has many inhabited islands with no neurointerventionalists. Our hospital on the mainland is a regional hub for eight island hospitals. We evaluated clinical outcomes of MT for patients with AIS on these islands versus on the mainland.

**Methods:**

During 2014–2019, we reviewed consecutive patients with AIS who received MT at our hospital. Patients comprised the Islands group and Mainland group. Patient characteristics and clinical outcomes were compared between groups.

**Results:**

We included 91 patients (Islands group: 15 patients, Mainland group: 76 patients). Seven patients (46.7%) in the Islands group versus 43 (56.6%) in the Mainland group achieved favorable outcomes. Successful recanalization was obtained in 11 patients (73.3%) on the islands and 67 (88.2%) on the mainland. The median OTP time in the Islands was 365 min. In both the Islands and Mainland groups, the OTP time and successful recanalization were associated with functional outcome. The modified Rankin Scale (mRS) score at 90 days ≤2 was obtained in two patients and mRS = 3 in four patients among eight patients with OTP time >6 h.

**Conclusions:**

Few patients with AIS on remote islands have received MT. Although patients who underwent MT on the islands had longer OTP, the clinical outcomes were acceptable. OTP time on remote islands must be shortened, as this is related to functional outcome. In some cases with successful recanalization, a favorable outcome can still be obtained even after 6 h. Even if OTP exceeds 6 h, it is desirable to appropriately select patients and actively perform MT.

## Introduction

Mechanical thrombectomy (MT) has become a standard treatment for acute ischemic stroke (AIS) with large-vessel occlusion (LVO) within 6 h of symptom onset to treatment initiation (groin puncture) (OTP) [1–3]. Per results of the recent DEFUSE 3 (Endovascular Therapy Following Imaging Evaluation for Ischemic Stroke) and DAWN (DWI or CT Perfusion Assessment with Clinical Mismatch in the Triage of Wake-Up and Late Presenting Strokes Undergoing Neurointervention with Trevo) trials, the therapeutic time window for MT has been extended to within 24 h of stroke onset for selected patients, based on imaging and clinical examinations [3–5]. The results of these studies indicate that patients with ischemic brain tissue that have not yet undergone infarction, or with penumbra, have improved functional outcomes with MT. The number of patients receiving MT has increased [6, 7], accounting for an estimated 6% of overall patients with AIS in 2018 in Japan [7]. However, MT treatment remains low in rural and remote areas [6].

**Fig 1:**
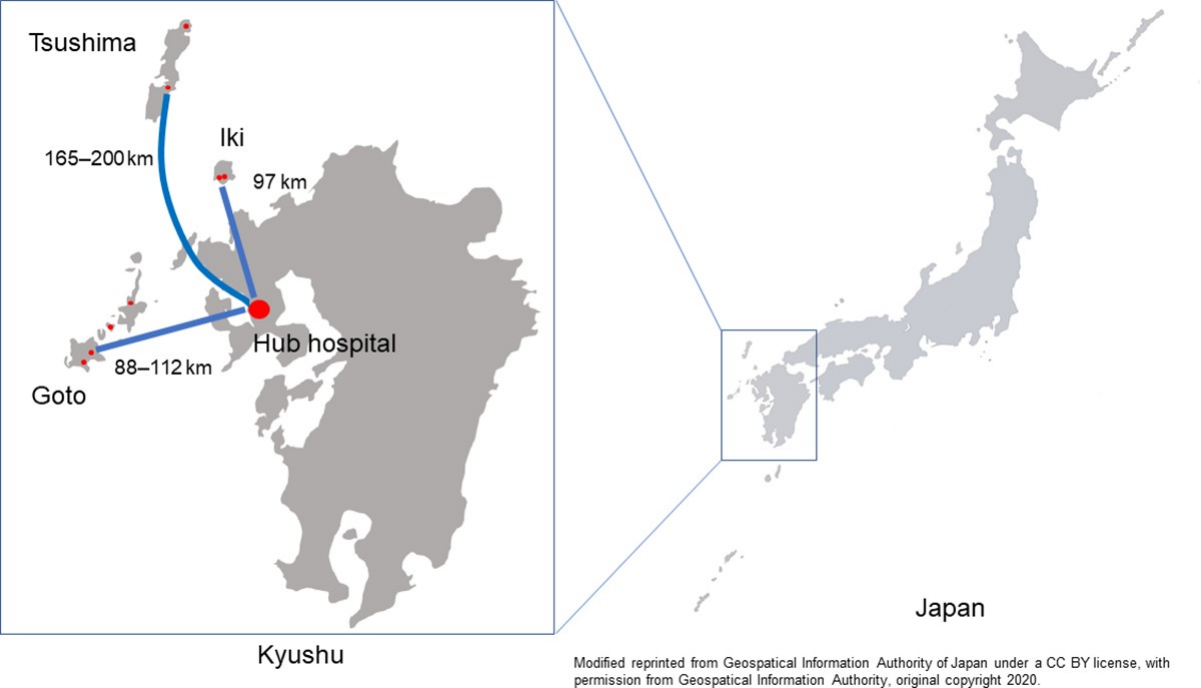
Geographic location of eight hospitals on islands and hub center in Nagasaki Prefecture, Kyushu, Japan. Modified reprint from the Geospatial Information Authority of Japan under a CC BY license, with permission from the Geospatial Information Authority, original copyright 2020.

Nagasaki Prefecture has 51 inhabited islands, the largest number in Japan, comprising three major island groups: Goto, Iki, and Tsushima (Fig 1). There are approximately 120,000 people living on these islands. However, there are no neurointerventionalists on the islands [8]. Our hospital, located on the mainland, serves as a regional hub for the island hospitals in Nagasaki Prefecture as well as local hospitals on the mainland. For the management of patients with AIS on these islands, we have established a helicopter transportation and teleradiology system that operates 24 h a day, 7 d/wk [8, 9].

The drip and ship (DS) method, whereby intravenous recombinant tissue plasminogen activator (IV rt-PA) is administered in a regional hospital, followed by patient transfer to a specialized stroke center, can facilitate early initiation of IV rt-PA [8, 10]. We previously reported that DS thrombolytic therapy is performed in six of the eight hospitals on the three main islands of Nagasaki [8, 9]. A nationwide study showed that the DS method could contribute to an increase in the rate of IV rt-PA; this method is currently used for one in four patients treated with IV rt-PA in the United States (US) [10]. For MT, several studies have also reported the safety and effectiveness of the drip, ship, and retrieve approach in AIS [11, 12].

In the present study, we clarified the clinical outcomes of MT in patients with AIS on islands who were transported to the hub center via the ship and retrieve (SR) method.

## Methods

### Patients

We reviewed the records of patients with AIS who underwent MT in our hub hospital between January 2014 and December 2019. The inclusion criteria for MT were as follows: (1) OTP within 6 h of stroke onset, (2) no evidence of intracranial hemorrhage (ICH), (3) M1 segment of the middle cerebral artery (M1), M2 segment of the middle cerebral artery (M2), internal cerebral artery (ICA), basilar artery (BA) on magnetic resonance (MR) angiography (MRA) or conventional angiography with clinical deficits. For patients with 6–24 h OTP, MT was indicated if there was an MR diffusion weighted imaging (DWI)-fluid-attenuated inversion recovery (FLAIR) mismatch [13, 14], that is, an acute ischemic lesion on DWI without marked parenchymal hyperintensity on FLAIR, in patients with M1, M2, ICA, or BA occlusion on MRA and clinical deficits. Patients with a pre-morbid modified Rankin Scale (mRS) score 3–5 were excluded in the present study.

**Fig 2:**
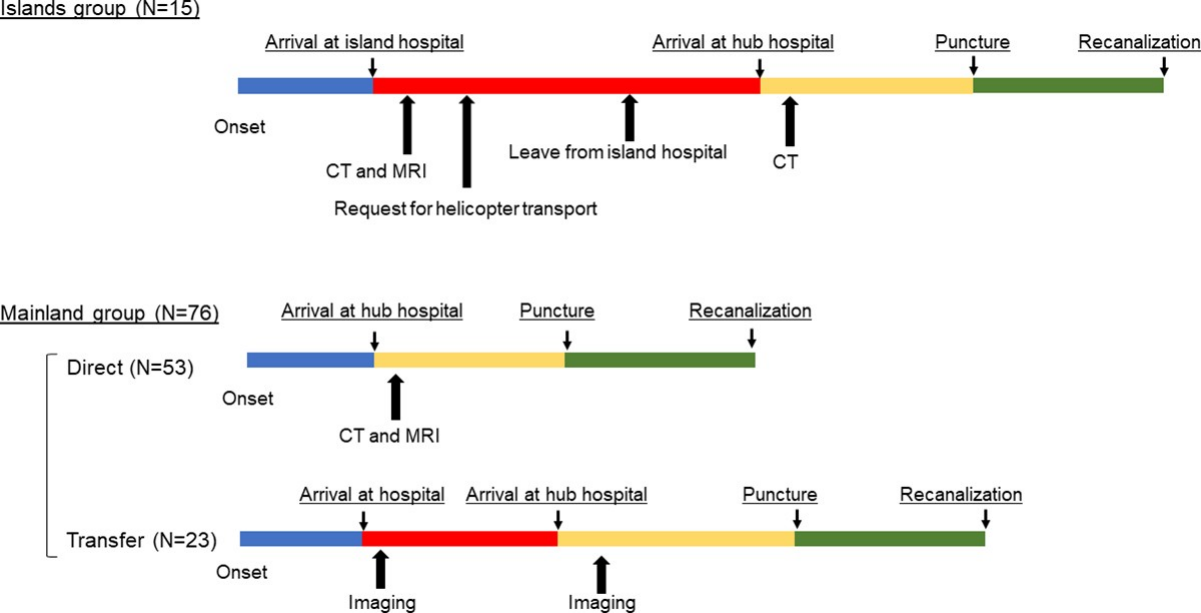
Comparison of time metrics for patients with acute ischemic stroke in the Islands and Mainland groups. The Mainland group was divided into two groups according to whether the patient was brought in directly (Direct) or was transferred from another hospital (Transfer). CT, computed tomography, MRI, magnetic resonance imaging.

We divided the included patients into two groups. Patients in the “Islands” group were transferred to our hospital from remote islands in Nagasaki Prefecture (Goto, Iki, and Tsushima); the “Mainland” group consisted of patients who lived on the mainland (Fig 2). IV rt-PA was performed following the criteria established by the Japan Stroke Society.

Patient demographic and baseline characteristics including age, sex, severity of neurological deficit using the National Institutes of Health Stroke Scale (NIHSS) score, and stroke subtype (according to TOAST [Trial of ORG 10172 in Acute Stroke Treatment] classification) [15] were compared between the two groups. We calculated the time between symptom onset and arrival at our hospital, groin puncture, and reperfusion as the onset-to-door (OTD), OTP, and onset-to-reperfusion (OTR) times, respectively. When the symptom onset time was unknown, the last known normal time was defined as symptom onset. Occlusion of the M1, M2, ICA, and BA was defined as LVO. Other variables included the reperfusion rate, based on the modified Thrombolysis in Cerebral Infarction (mTICI) grade [16], mRS score at 90 days after stroke onset, and adverse event incidence. Hemorrhagic transformation (HT) was defined as any ICH in the territory of the initial infarction during hospitalization and was confirmed using multiple non-contrast head computed tomography (CT) or MR imaging (MRI). HT was categorized as a hemorrhagic infarction, type 1 or type 2 (HI-1 or HI-2) or parenchymal hematoma, type 1 or 2 (PH-1 or PH-2), according to definitions of the European Cooperative Acute Stroke Study [17]. Symptomatic ICH was defined as neurological deterioration of 4+ points in the NIHSS score within 24 h post procedure.

### Ethics statement

This study was approved by the ethics committee of National Hospital Organization Nagasaki Medical Center (approval number 28078). This was a retrospective study of medical records, and the data were analyzed anonymously. All clinical investigations described in this study were conducted in accordance with the principles expressed in the Declaration of Helsinki.

### Teleradiology system and helicopter system

Our hospital, located on the mainland, serves as a hub hospital for eight hospitals on the islands of Nagasaki Prefecture, Kyushu, Japan (Fig 1). The SYNAPSE Teleradiology system (Fujifilm) connects our hospital with hospitals on the islands via Ajisai-net, which is the Japanese Health Information Exchange in Nagasaki [8]. In addition, a new information and communication technology (SYNAPSE ZERO; Fujifilm) using smart devices was introduced into the teleradiology system [9]. In the Islands group, a physician conducted a neurological assessment based on the NIHSS score at admission to an island hospital. Non-enhanced head CT and MRI data of all patients with AIS on the islands were then transmitted via the teleradiology system. The patients were transferred to our hub hospital using the SR method (Fig 2, Islands group). Most patients with AIS who had LVO on the remote islands, without rt-PA and/or MT treatment, were managed at hospitals on the islands; these cases were not included in this study. The teleradiology system was not used in the Mainland group.

Our hospital uses three types of helicopter: helicopter emergency medical services (HEMS), a Maritime Self-Defense Force (MSDF) helicopter, and a firefighting disaster prevention (FDP) helicopter. Characteristics of each helicopter have been detailed in a previous report [8].

### Endovascular treatment

Patients underwent MT using a suction catheter (Penumbra System; Penumbra, Alameda, CA, USA) and/or stent retriever (Solitaire FR; Medtronic Neurovascular, Irvine, CA, USA and Trevo ProVue; Stryker Neurovascular, Fremont, CA, USA) under local anesthesia. Other procedures included aggressive clot disruption with a microwire, balloon angioplasty, or carotid stenting.

### Evaluation of clinical outcomes

Based on the mRS score at 90 days after stroke onset, each patient’s clinical outcome was evaluated as either “favorable” or “unfavorable” for scores ≤ 2 or ≥ 3, respectively.

### Statistical analysis

The aim of the present study was to summarize the current status of our helicopter transportation system; we therefore present inference statistics as descriptive ones [18] rather than inference of the larger population. A cumulative distribution function of time was used to evaluate time-to-event variables. The effect of each factor on the distributional difference for each variable of interest was described using *p*-values obtained from the normal asymptote of the Mann–Whitney U test. Dependency between two binomials was described with a *p*-value from the chi-square distribution of an appropriate degree of freedom. Analyses were performed using IBM SPSS, version 25.0 (IBM Corp., Armonk, NY, USA), JMP 14.2 (SAS Institute, Cary, NC, USA), and R version 4.1.0. (The R Project for Statistical Computing, Vienna, Austria) [19].

## Results

### Patient characteristics

Among 112 patients treated with MT, 21 patients with a pre-morbid modified Rankin Scale (mRS) score 3–5 were excluded. Thus, 91 patients were included in this study, with 15 patients in the Islands group and 76 patients in the Mainland group. In the Mainland group, 53 patients were delivered to the hub hospital directly and 23 patients were transferred to the hub hospital (Fig 2, Mainland group). IV rt-PA was performed in 8 patients (53.3%) in the Islands group and 32 patients (42.1%) in the Mainland group (Table 1). The median patient age was 72.0 years in the Islands group and 78.0 years in the Mainland group (Table 1). Among the 91 patients, there were no marked differences between the two groups regarding the initial NIHSS score and distribution of stroke subtype.

**Table 1:** Baseline characteristics of patients treated with mechanical thrombectomy in the Islands and Mainland groups.

	Total	Islands	Mainland	*p* value
Number of patients	91	15	76	
Male sex, n (%)	56 (61.5)	8 (53.3)	48 (63.2)	0.671
Age (years); median (IQR)	78.0 (66.0–82.0)	72.0 (63.5–83.0)	78.0 (67.8–82.0)	0.422
Hypertension, n (%)	58 (63.7)	9 (60.0)	49 (64.5)	0.972
Hyperlipidemia, n (%)	15 (16.5)	4 (26.7)	11 (14.5)	0.434
Diabetes mellitus, n (%)	19 (20.9)	2 (13.3)	17 (22.4)	0.729
Smoking, n (%)	12 (13.2)	1 (6.7)	11 (14.5)	0.682
Atrial fibrillation, n (%)	34 (37.4)	3 (20.0)	31 (40.8)	0.155
Helicopter transport, n (%)	32 (35.2)	15	17 (22.4)	<0.001
Transfer, n (%)	38 (41.8)	15	23 (30.3)	<0.001
Time from symptom onset to door at our hospital (OTD) (min); median (IQR)	166 (58–323)	321 (273–659)	135 (53–279)	<0.001
Time from symptom onset to puncture (OTP) (min); median (IQR)	270 (162–373)	365 (317–728)	235 (169–361)	0.005
Time from symptom onset to recanalization (OTR) (min); median (IQR)	346 (274–440)	426 (396–861)	321 (261–426)	0.005
Initial NIHSS score; median (IQR)	17.0 (13.0–23.0))	18.0 (14.5–23.0)	16.5 (13.0–23.0)	0.768
Occlusion site				0.789
ICA	21	5	17	
M1	35	6	32	
M2	24	4	18	
BA	9	0	9	
TOAST classification				
Cardioembolism, n (%)	57 (62.6)	7 (46.7)	50 (65.8)	0.268

IQR, interquartile range; NIHSS, National Institutes of Health Stroke Scale; ICA, internal carotid artery; M1, M1 segment of the middle cerebral artery; M2, M2 segment of the middle cerebral artery; BA, basilar artery; TOAST, Trial of ORG 10172 in Acute Stroke Treatment.

All patients in the Islands group were transported via helicopter. Three types of helicopter were used in the Islands group: HEMS (nine patients), an MSDF helicopter (five patients), and an FDP helicopter (one patient) (Table 2). The time from arrival at hospitals on the islands to arrival at the hub hospital was longer for patients who were transported with an MSDF/FDP helicopter than with HEMS (the median of the respective groups was 272 min and 220 min for MSDF/FDP and HEMS, respectively). The CT-Alberta Stroke Program Early CT score (ASPECTS) decreased during patient transport from the islands to the hub hospital (Table 2). In the Islands group, the OTD time was within 6 h in 10 patients (66.7%) and within 6–24 h in 5 patients (33.3%). Helicopter transport was used for 17 of 76 patients in the Mainland group (Table 1), and all these patients were transported using HEMS. The median transport distance from the islands to the hub hospital was 112 km (interquartile range [IQR]: 92.5–112), and the transport distance by HEMS in the Mainland group was 22 km (IQR: 9.0–33.0). Fourteen patients in Goto and one patient in Iki received MT; no patients in Tsushima underwent MT (Table 2). Tsushima is the furthest island group from the mainland among the islands included in this study. Among eight hospitals on the islands, patients receiving MT were limited to those who were transported from only three hospitals on the islands. The median OTP time in the Islands group was 365 min (IQR: 317–728). The OTD, OTP, and OTR times in the Islands group were longer than those in the Mainland group; the median [IQR] of OTD, OTP, and OTR times was 321 min [273–659], 365 min [317–728], and 426 min [396–861]; and 135 min [53–279], 235 min [169–361], and 321 min [261–426] for the Islands and Mainland groups, respectively.

**Table 2:** Baseline characteristics of patients treated with ship and retrieve method on the Islands.

Number of patients	15
Distance (km); median (IQR)	112 (92.5–112)
Patients from each island group, n	
Goto	14
Iki	1
Tsushima	0
Type of helicopter transport, n	
HEMS	9
Firefighting disaster prevention helicopter	1
JMSDF helicopter	5
Time from symptom onset to door at island hospitals (min); median (IQR)	55 (30–99)
Time from island hospital door to door at hub hospital (min); median (IQR)	242 (215–273)
ASPECTS; median (IQR)	
At island hospitals	9.0 (8.5–10.0)
At hub hospital	8.0 (7.0–8.0)
MRI DWI ASPECTS (at isolated island hospitals); median (IQR)	8.0 (7.0–8.8)

IQR, interquartile range; HEMS, helicopter emergency medical services; JMSDF, Japan Maritime Self-Defense Force; CT, computed tomography; ASPECTS, Alberta Stroke Program Early CT score; MRI, magnetic resonance imaging; DWI, diffusion-weighted imaging.

In the Mainland group, the median OTP time in the Direct group was longer than that in the Transfer group; the median [IQR] of OTP time was 215 min [140–300]; and 311 min [280–377] for the Direct and Transfer groups, respectively (data not shown).

### Functional outcomes

**Fig 3:**
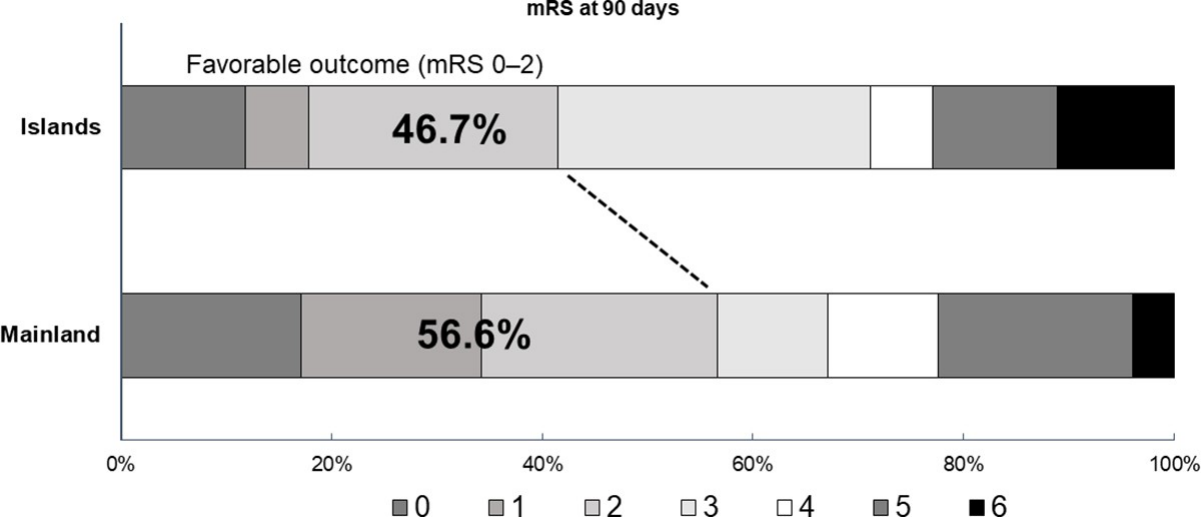
Distribution of mRS score at 90 days in patients with acute ischemic stroke receiving mechanical thrombectomy in the Islands and Mainland groups. In the Islands group, 7 of 15 patients (46.7%) achieved favorable outcomes (mRS score ≤2) whereas 43 of 76 patients (56.6%) in the Mainland group had favorable outcomes. mRS, modified Rankin Scale.

**Fig 4:**
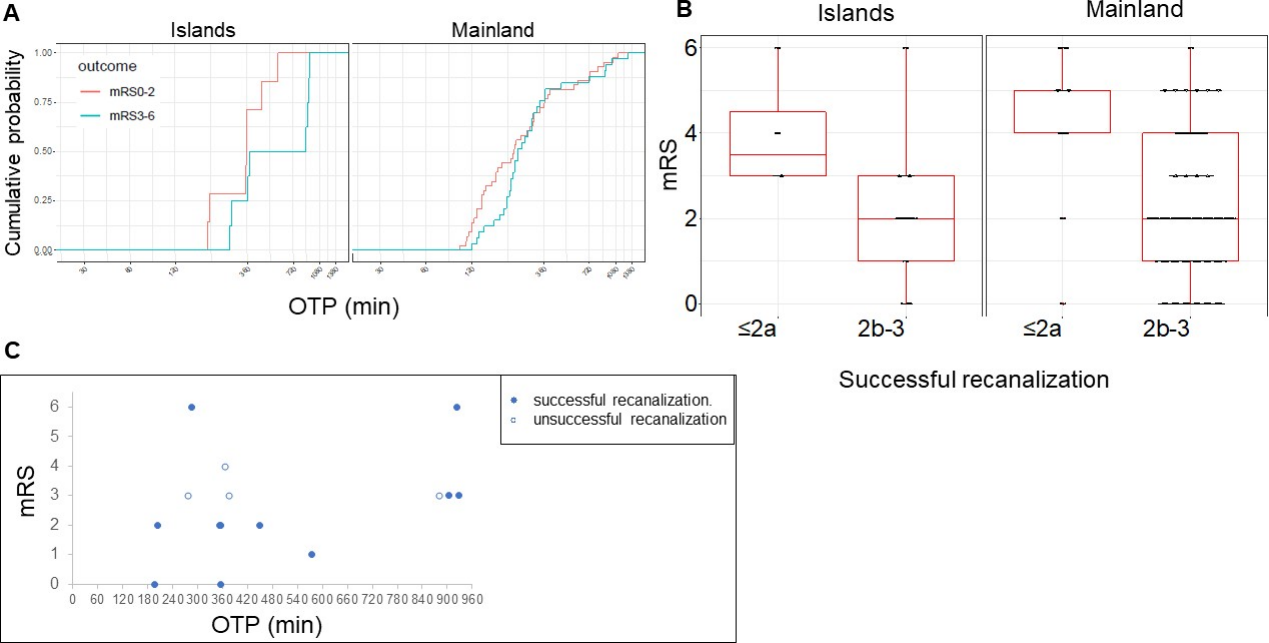
Functional outcomes and OTP time in the Islands and Mainland groups. (A) OTP time was related to functional outcome in both the Islands and Mainland groups. Distribution of the mRS score at 90 days and successful recanalization in the Islands and Mainland groups (B) Successful recanalization (modified Thrombolysis in Cerebral Infarction [mTICI] grade ≥2b) was related to functional outcome in both the Islands and Mainland groups. (C) Closed circle: successful recanalization. Open circle: unsuccessful recanalization (mTICI <2b). mRS ≤2 was found in five of seven patients with an OTP time ≤360 min; two patients had mRS ≤2 and four had mRS = 3 among eight patients with an OTP time >360 min. OTP, onset to puncture, mRS, modified Rankin Scale.

Symptomatic ICH was present in 1 of 15 patients (6.7%) in the Islands group and in 3 of 76 patients (3.9%) in the Mainland group (Table 3). In the Islands group, 7 of 15 patients (46.7%) achieved favorable outcomes (mRS score ≤2) whereas 43 of 76 patients (56.6%) in the Mainland group had favorable outcomes (Fig 3). The cumulative probability function of OTP stratified by the mRS score at 90 days after stroke onset (Fig 4A) suggested consistency across the Islands and Mainland groups with respect to shorter time of OTP being more likely to result in a favorable functional outcome (defined in the Methods). In the Islands group, mRS score at 90 days ≤2 was found in five of seven patients with OTP time ≤6 h whereas two patients had mRS ≤2 and four patients had mRS = 3 among eight patients with an OTP time >6 h (Fig 4C).

**Table 3:** Clinical results of patients treated with mechanical thrombectomy in the Islands and Mainland groups.

	Total	Islands	Mainland	*P* value
Number of patients	91	15	76	
IV rt-PA, n (%)	40 (44.0)	8 (53.3)	32 (42.1)	0.606
Successful recanalization (mTICI 2b, 3), n (%)	78 (85.7)	11 (73.3)	67 (88.2)	0.273
sICH, n (%)	4 (4.4)	1 (6.7)	3 (3.9)	0.520
Hemorrhagic transformation, n (%)	27 (29.7)	6 (40.0)	21 (27.6)	0.516
HI-1	3	3	0	
HI-2	14	3	11	
PH-1	7	0	7	
PH-2	3	0	3	
mRS ≤2 at 90 days, n (%)	50 (54.9)	7 (46.7)	43 (56.6)	0.674
Mortality during admission, n (%)	5 (5.5)	2 (13.3)	3 (3.9)	0.189

IV rt-PA, intravenous recombinant tissue plasminogen activator; mTICI, modified Thrombolysis in Cerebral Infarction; sICH, symptomatic intracranial hemorrhage; HI, hemorrhagic infarction; PH, parenchymal hemorrhage; mRS, modified Rankin Scale.

Successful recanalization (mTICI ≥2b) was obtained in 11 of 15 patients (73.3%) on the islands and 67 of 76 patients (88.2%) on the mainland (Table 3). Successful recanalization was related to functional outcome in both the Islands and Mainland groups (Fig 4B). In the Islands group, successful recanalization was obtained in both patients with mRS score ≤2 at 90 days and with an OTP time >6 h. All four patients with unsuccessful recanalization (mTICI <2b) in the Islands group had unfavorable outcomes (Fig 4B and 4C).

## Discussion

During 2014–2019, only 15 patients on the remote islands of Nagasaki Prefecture underwent MT, with no patients from Tsushima. The DR approach (IV rt-PA) was performed in 61 patients at six of eight hospitals on the islands during the same period [8, 9]. There were very few patients with AIS on the islands who underwent MT in comparison with IV rt-PA. The Recovery by Endovascular Salvage for Cerebral Ultra-acute Embolism (RESCUE)-Japan Project reported that MT for AIS was performed in 9.8 patients per 100,000 population in Japan during 2018 [20]. There are potentially more MT-adapted cases on the islands.

In the Islands group, 7 patients (46.7%) achieved favorable outcomes (mRS score ≤2) whereas 43 (56.6%) in the Mainland group achieved favorable outcomes. The median OTR time in the Islands group was 426 min (IQR: 396–861). The clinical results of function outcome in the Islands group was acceptable, in comparison with the results of randomized control trials [21].

The median time from arrival at an island hospital to arrival at the hub hospital was 242 min (IQR: 215–273). Interhospital transfers can exceed 2 h even in high-volume primary stroke centers [22]. Patients with AIS living on remote islands are first delivered to hospitals on an island and are then transferred to the hub hospital via helicopter transport in selected cases. A certain amount of time is required for transport because patients on the islands are transported across the sea. In previous studies, the OTR time was found to affect the prognosis of patients [23]. It is therefore important to reduce the time of delivery of patients with AIS to a hub center. Another possibility is to create a staff position for a doctor who can perform MT on remote islands, as some island hospitals have an angiography suite.

In the Mainland group, 30.3% of patients were secondarily transferred to our hub center and underwent MT. The median OTP time in the Direct group was longer than that in the Transfer group. Froehler et al. reported that secondary transfer leads to a considerable MT treatment delay and less likelihood of functional outcome, as compared with patients who are brought directly to a comprehensive stroke center [24]. Several clinical scales for predicting LVO have been reported [25]. Use of these scales may increase the number of patients treated directly and improve the functional outcome of MT.

In the Islands group, the proportion of patients achieving functional outcomes with an OTP time ≤6 h was high; however, even beyond 6 h, mRS score 0–3 at 90 days was observed in five of eight patients. This shows that even if the OTP time exceeds 6 h, the outcome can still be good, even on isolated islands. On the basis of the results of recent trials, the therapeutic time window for MT has been extended to within 24 h [4, 5]. DWI-FLAIR mismatch was associated with a more moderate outcome in patients receiving MT within 24 h [14]. For MT, time-based strategies are undergoing a transition to imaging-based strategies [26]. Thus, it is necessary to inform island hospitals that the indications for MT are expanding. When performing MT in patients on remote islands, we need to consider an imaging-based strategy. In the near future, more patients living on isolated islands, including Tsushima, might be able to receive MT.

Recently, robotic-assisted angiography has been applied in percutaneous coronary intervention (PCI) [27] and the interventional management of extracranial vascular disease [28]. Remote tele-robotic-PCI with an operator located 20 miles from the patient has been successfully performed [29]. In the future, such robotic technology may dramatically change MT for patients with AIS living in remote areas, freeing these patients from the need for helicopter transport from areas where there are no neurointerventionalists, such as on Nagasaki’s islands.

### Limitations

This study has several limitations. First, this was a retrospective, single-center study with a small number of patients. Second, we did not include patients with LVO managed without MT. Third, perfusion imaging was unavailable at our hospital and island hospitals in this study. In the future, a larger prospective study will be needed, to evaluate differences between AIS patients on remote islands and those on the mainland, treated with MT.

## Conclusions

Few patients with AIS living on remote islands in Nagasaki Prefecture have received MT. In our study, although patients who underwent MT in the island group had longer OTP, the clinical outcomes were acceptable. The OTP time on remote islands must be shortened, as this factor is related to functional outcome. In some cases with successful recanalization, a favorable outcome can still be obtained even after 6 h. Even if OTP exceeds 6 h, it is desirable to appropriately select patients and actively perform MT. We must increase the number of patients with AIS who are able to receive MT on these remote islands.
